# H_2_O_2_-GA_3_-Na_2_WO_4_ Synergistically Promotes Germination of Immature Winter Wheat Grains for Speed Breeding

**DOI:** 10.3390/plants15091313

**Published:** 2026-04-24

**Authors:** Dong Yan, Pengcheng Lv, Lichao Zhang, Dengke Wang, Tianyi Chen, Zefu Lu, Jizeng Jia, Lifeng Gao

**Affiliations:** State Key Laboratory of Crop Gene Resources and Breeding, Institute of Crop Sciences, Chinese Academy of Agricultural Sciences, Beijing 100081, China; 18500830907@163.com (D.Y.); lpc991205@163.com (P.L.); zhanglichao@caas.cn (L.Z.); wdk974753956@163.com (D.W.); C18568174471@163.com (T.C.); luzefu@caas.cn (Z.L.)

**Keywords:** immature wheat grains, germination promotion, chemical reagent combination, speed breeding

## Abstract

Seed germination is a critical initial stage of the plant life cycle, regulated by signaling pathways such as phytohormones and reactive oxygen species (ROS). However, the low germination rate of immature grains is a key bottleneck limiting wheat speed breeding. This study used immature grains of the winter wheat cultivar Kenong 199 (KN199) collected 18 days post anthesis to establish an efficient germination protocol. By screening individual and combined treatments of hydrogen peroxide (H_2_O_2_, 1%), gibberellin (GA_3_, 20 μM), and varying concentrations of abscisic acid (ABA) synthesis inhibitor sodium tungstate (Na_2_WO_4_), alongside transcriptome analysis, we identified the optimal reagent combination and gained preliminary insight into its molecular basis. The triple reagent combination of 0.5 mM Na_2_WO_4_ + 20 μM GA_3_ + 1% H_2_O_2_ exhibited the highest germination rate of 80%, approximately sevenfold higher than single reagent treatments, with germination rate peaking after 4 days. Transcriptome profiling revealed that this combination modulated the expression of key genes related to dormancy release and germination, including upregulation of GA biosynthesis gene *GA3ox2* and ABA catabolism gene *TaCYP707A2*, and downregulation of ABA biosynthesis and signaling genes (*ABI5*, *TaNCED1*, etc.). Additionally, genes associated with energy metabolism and transport pathways were enhanced. This optimized reagent combination significantly improves immature grain germination, shortens the breeding cycle, and provides a practical tool for achieving “five generations per year” speed breeding in winter wheat. Our findings contribute to seed biology by offering a chemical strategy to overcome dormancy in immature cereal grains.

## 1. Introduction

Seed germination is crucial for the plant life cycle and is regulated by various internal and external signals. The appropriate time to break dormancy, germinate, and establish a young seedling is essential for the propagation of the next generation [1]. Recently, several review articles have summarized the biochemical and molecular mechanisms underlying seed germination [1,2,3]. Phytohormone networks and reactive oxygen species (ROS) signaling are core to regulating seed dormancy and germination. Abscisic acid (ABA) is a central negative regulator of seed germination, whereas gibberellin (GA) plays a pivotal role in promoting seed germination [4]. GAs and ABA interact to regulate germination at the metabolic and signaling levels, with mutual involvement in suppressing each other’s biosynthesis [5]. A high ABA:GA ratio maintains dormancy, while a low ratio releases it. Hormonal balance regulation involves complex signaling crosstalk. Sodium tungstate (Na_2_WO_4_), as an ABA synthesis inhibitor, was reported to inhibit the activity of abscisic aldehyde oxidase and lower the formation of ABA in barley seedlings [6]. Abscisic acid insensitive 5 (ABI5) acts as a hub in the ABA–GA antagonism during seed germination [1]. As a transcription factor, ABI5 directly regulates the expression of genes involved in ABA and GA signaling, ultimately mediating the levels of both phytohormones and/or their signals. Besides ABA and GA, other phytohormones (e.g., auxins, ethylene, and salicylic acid) also participate in regulating seed dormancy and germination [3].

ROS act as second messengers with dual roles in seed germination: low levels maintain dormancy, high levels cause seed deterioration, and appropriate levels trigger the transition from dormant to germinating seeds [7]. During seed germination, ROS can directly interact with cell wall polysaccharides, potentially promoting radicle cell elongation [8]. Additionally, following water imbibition, endosperm weakening—a key step in initiating germination—is controlled by the crosstalk of ROS–GA–ABA signaling pathways [3]. A recent study on primed tomato seeds showed that H_2_O_2_ enhances germination capacity by reducing the ABA/GA3 ratio via upregulating the expression of GA biosynthesis gene *GA3ox1* and ABA catabolism gene *ABA 8-hydroxylase* [9]. Also, H_2_O_2_ was used to break the dormancy of wheat seeds harvested at 35 days post anthesis (dpa) [10]. These results confirm that ROS positively regulate GA synthesis and ABA degradation, thereby facilitating dormancy release and germination.

Wheat (*Triticum aestivum* L.) is a major global food crop, and its yield is directly linked to global food security. In recent years, breakthroughs and applications in speed breeding and CRISPR-based genome editing technologies have significantly advanced the genetic improvement of wheat. However, the long seed maturation cycle and inherent physiological dormancy remain key bottlenecks limiting breeding efficiency. The developmental stages of wheat grains can be divided according to grain phenotypes into grain formation (1–4 dpa), grain enlargement or early milk stage (4–10 dpa), milk stage (11–16 dpa), soft dough stage (17–21 dpa), hard dough stage (22–30 dpa), and maturity or fully ripe stage (55 dpa) [11]. The period from 14 to 28 dpa is the most active phase for wheat grain filling [12]. Among these, around 18 dpa is a critical point: at this stage, cell division in the sub-aleurone layer nearly ceases, the embryo structure is relatively well-developed, and grain filling enters a phase of linear rapid growth [12]. Additionally, 11–15 dpa is also a key period for starch synthesis and accumulation in seeds [13]. Gudi et al. [14] proposed strategies to accelerate germination, and Ghosh et al. [15] reported the use of GA_3_ to break seed dormancy. Nevertheless, there is a lack of practical and efficient technical systems for promoting the germination of immature winter wheat grains, and the relevant supporting gene expression characteristics have not been supplemented and analyzed.

In this study, we used H_2_O_2_, GA_3_ and Na_2_WO_4_ to address this bottleneck, and aimed to: (1) determine the optimal chemical reagent combination to maximize immature grains germination; and (2) preliminarily reveal the transcriptional regulatory characteristics of the Na_2_WO_4_ + GA_3_ + H_2_O_2_ combination in breaking seed dormancy via transcriptome analysis. The established protocol enables the direct utilization of immature grains in wheat speed breeding and provides practical technical support for winter wheat genetic improvement.

## 2. Results

### 2.1. Screening of Optimal Reagent Combination for Promoting Germination of Immature Wheat Grains

**Figure 1:**
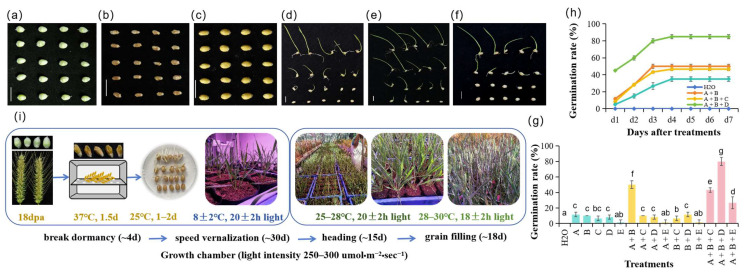
Morphology of immature wheat grains and their germination performance under different reagent treatments in speed breeding conditions. (**a**) Immature wheat grains at 18 dpa; (**b**) immature grains dried at 37 °C for 36 h; (**c**) dried immature grains after 24 h imbibition in ultrapure water; (**d**) germination of grains after 6 d treatment with GH (20 μM GA_3_ + 1% H_2_O_2_); (**e**) germination of grains after 6 d treatment with WGH (0.5 mM Na_2_WO_4_ + 20 μM GA_3_ + 1% H_2_O_2_); (**f**) germination of grains after 6 d treatment with (1 mM Na_2_WO_4_ + 20 μM GA_3_ + 1% H_2_O_2_). Scale bar = 1 cm. (**g**) Germination rate 3 days after treatment. Different lowercase letters indicate significant differences among treatments (one-way ANOVA, SPSS v27, *p* < 0.05). (**h**) Daily germination kinetics over seven days. In (**g**,**h**), reagent and combination are shown in Table S1, and the treatment code is A: 1% H_2_O_2_; B: 20 μM GA3; C: 0.2 mM Na_2_WO_4_; D: 0.5 mM Na_2_WO_4_; E: 1 mM Na_2_WO_4_. Values are mean ± SD (*n* = 60). (**i**) Schedules for five generation cycles of winter wheat under speed breeding.

The 18 dpa immature wheat grains (early soft dough stage) showed light green seed coats and plump morphology (Figure 1a) and had basic germination potential. After drying at 37 °C for 36 h, the grain moisture content decreased sharply, the seed coats turned yellowish-brown, and the grains shrank (Figure 1b), and their physical traits recovered to a state similar to mature grains after water imbibition (Figure 1c).

Ultrapure water was used as the control, and the germination rates of immature grains under the single reagent (H_2_O_2_, GA_3_, or Na_2_WO_4_), two-reagent (H_2_O_2_ + GA_3_, H_2_O_2_ + Na_2_WO_4_, or GA_3_ + Na_2_WO_4_), and triple-reagent (H_2_O_2_ + GA_3_ + Na_2_WO_4_) combinations were determined (Table S1). No germination was observed in the control group (Figure 1g,h), and significant differences were found in the germination-promoting effects of different treatments.

Single reagent treatments showed limited germination-promoting effects: 1% H_2_O_2_ and 20 μM GA_3_ had the best effects, with germination rates of only 12% and 10%, respectively; 0.2 mM and 0.5 mM Na_2_WO_4_ had a lower effect (7% and 8%); and 1 mM Na_2_WO_4_ had almost no germination-promoting effect (Figure 1g).

The germination rates of two-reagent combinations varied significantly: the combination of 20 μM GA_3_ + 1% H_2_O_2_ (hereinafter referred to as GH) achieved a germination rate of 50%, which was significantly higher than other two-reagent combinations (*p* < 0.01) (Figure 1d,g); the combination of 1% H_2_O_2_ or 20 μM GA_3_ with 0.2 mM or 0.5 mM Na_2_WO_4_ had no significant germination-promoting effect; and the combination with 1 mM Na_2_WO_4_ showed no germination (Figure 1g), indicating that high concentrations of Na_2_WO_4_ inhibits germination of immature grains.

Based on the GH two-reagent combination, 0.2 mM, 0.5 mM, or 1 mM Na_2_WO_4_ were added to form triple-reagent combinations for further screening. The combination of 0.5 mM Na_2_WO_4_ + 20 μM GA_3_ + 1% H_2_O_2_ (hereinafter referred to as WGH) achieved the highest germination rate of 80%, which was significantly higher than the GH combination and other triple-reagent combinations (*p* < 0.01) (Figure 1e,g,h); the triple-reagent combination with 1 mM Na_2_WO_4_ still had the lowest germination rate (27%), further confirming the inhibitory effect of high concentration Na_2_WO_4_ (Figure 1f–h).

Continuous 7 day monitoring showed that the germination rates of the GH and WGH groups both reached a plateau 4 days after treatment (Figure 1h). The GH combination increased the germination rate fourfold, while WGH increased by sevenfold compared with the single reagent treatment. WGH was the optimal reagent combination for promoting the germination of 18 dpa immature wheat grains. This result suggests that in speed breeding practice, sowing can be carried out 3 days after reagent treatment of immature grains, which effectively shortens the breeding cycle (Figure 1i).

### 2.2. Transcriptome Analysis of the Optimal Reagent Combination Promoting Immature Wheat Grain Germination

To supplementally explore the gene expression characteristics of WGH combination in promoting the germination of immature wheat grains, transcriptome sequencing was performed on WGH- and GH-treated grains at radicle emergence, with water-imbibed grains as the control. The sequencing data had high quality and sufficient depth (Table S2, Figure S1), which provided a basis for subsequent analysis.

Comparative analysis showed that there were 13,148 differentially expressed genes (DEGs) (FDR < 0.05, |log2(fold change)| ≥ 1) in the GH group and 11,697 DEGs in the WGH group compared with the control (Figure S2a,b). A total of 10,733 DEGs were shared between the two groups, with 2415 and 964 being group-specific to GH and WGH, respectively. Functional enrichment analysis indicated that these DEGs were mainly associated with key biological processes related to seed germination, including dormancy termination, embryonic cell cycle re-initiation, cell wall formation and energy supply (Figure S3a,b). Consistent with the germination-promoting effect, the expression of late embryogenesis abundant (LEA) protein genes was significantly downregulated (Figure S4a,b). LEA genes are related to seed maturation and dehydration tolerance [16,17], and the downregulation may reflect the loss of desiccation tolerance of immature grains during the germination process. The expression of starch degradation-related genes that are involved in energy supply was upregulated. Twenty α-amylase-encoding genes showed higher expression in the WGH group than in the GH group (Table S3), which was consistent with the higher germination rate of the WGH group.

**Figure 2:**
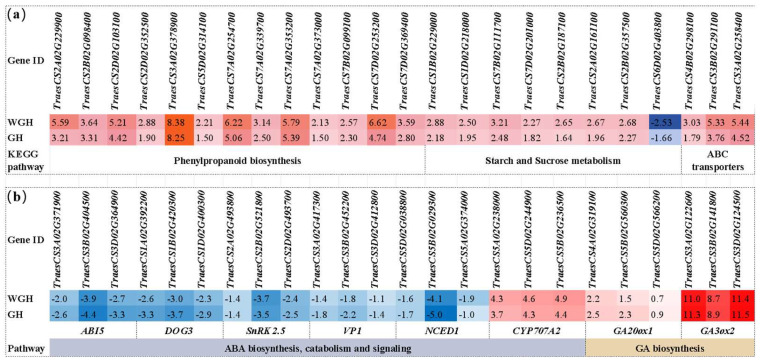
Differentially expressed genes (DEGs) response to WGH treatment. (**a**) Genes specially responsive to WGH treatment, including those involved in phenylpropanoid biosynthesis, starch and sucrose metabolism and ABC transporter pathways; (**b**) expression changes in core genes involved in ABA and GA metabolism and signaling under GH and WGH treatments relative to the water control. Values are log_2_(FoldChange). Red indicates upregulation and blue indicates downregulation compared with the water control. WGH treatment consists of 0.5 mM Na_2_WO_4_, 20 μM GA_3_ and 1% H_2_O_2_, while GH consists of 20 μM GA_3_ and 1% H_2_O_2_.

A total of 964 WGH-specific DEGs (778 upregulated, 186 downregulated) were identified by comparing the WGH and GH groups. GO and KEGG enrichment analysis showed that these genes were mainly enriched in catalytic activity, membrane transport, metabolic processes, plant hormone signal transduction and phenylpropanoid biosynthesis (Figure S5a,b). Notably, 13 phenylpropanoid biosynthesis-related genes and three ABC transporter pathway-related genes were significantly upregulated in the WGH group (Figure 2a), which may be related to the enhanced germination-promoting effect of the triple-reagent combination.

### 2.3. Expression Characteristics of Key Hormone Regulation Genes Under the Optimal Reagent Combination

ABA/GA balance is the core of seed germination regulation, and *ABI5* is a key hub gene in the ABA–GA antagonistic pathway [1]. Preliminary transcriptome analysis showed that the WGH combination significantly altered the expression of key genes in ABA–GA signal crosstalk (Figure 2b): genes related to ABA biosynthesis and signaling (*ABI5*, *TaNCED1*, *TaVP1*, *TaSnRK2.5* and *TaDOG3*) were significantly downregulated, while ABA catabolism gene *TaCYP707A2* and GA biosynthesis genes *GA3ox2* and *GA20ox1* were significantly upregulated. The above gene expression changes indicate that the optimal reagent combination can regulate the ABA/GA balance at the transcriptional level, which is one of the important characteristics of its promotion of immature grain germination.

## 3. Discussion

Through screening and optimizing chemical reagent combinations, this study successfully achieved efficient germination of immature grains of winter (facultative winter) wheat at 18 days after anthesis, with the highest germination rate over 80%. The reagent combination provides key technical support for breaking the breeding cycle limitation using immature grains and ensuring the five generations per year speed breeding of winter (facultative winter) wheat.

### 3.1. The Synergistic Effect of the Optimal Reagent Combination Is the Key to Improving the Germination Rate of Immature Grains

Immature grains are usually difficult to germinate directly due to incomplete embryo development, accumulation of inhibitory substances, or seed coat barriers. In this study, a single reagent such as 1% H_2_O_2_ or 20 μM GA_3_ could promote germination, but with limited effects (less than 10%); however, their combined use (20 μM GA_3_ + 1% H_2_O_2_) increased the germination rate to 50% (Figure 1g), showing a significant synergistic effect: H_2_O_2_ may break the seed coat barrier or activate embryo vitality through oxidative stress [8], and GA_3_ promotes embryo elongation and nutrient mobilization [5]. On this basis, adding an appropriate concentration of Na_2_WO_4_ (0.5 mM) further optimized the germination effect, increasing the germination rate to 80% (Figure 1g,h). Na_2_WO_4_, as an ABA synthesis inhibitor [6], may reduce the content of endogenous inhibitory ABA in immature grains by suppressing its biosynthesis. Under WGH treatment, we observed the downregulation of ABA synthesis gene *TaNCED1* and the upregulation of ABA catabolic gene *TaCYP707A2*, which may optimize the hormonal balance and form a synergistic germination-promoting effect with H_2_O_2_ and GA_3_. A high concentration of Na_2_WO_4_ (1 mM) showed an inhibitory effect on germination, which may be due to the excessive interference of endogenous hormone balance and normal metabolic processes of grains, indicating that the concentration of Na_2_WO_4_ is a key technical parameter of this germination technology. However, it should be noted that tungsten, as a molybdenum analog, may inhibit the activity of molybdenum-dependent enzymes such as nitrate reductase [18]. The exogenous application of tungstate could therefore potentially affect nitrogen assimilation and subsequent plant development. In this study, seedlings derived from WGH-treated seeds exhibited normal morphology and growth at the early stage. Nevertheless, the impact on the entire growth cycle and yield requires further evaluation for optimizing the protocol.

Transcriptome analysis supplemented the gene expression characteristics by which the WGH combination synergistically promotes immature grain germination: the combination regulated the expression of key genes related to ABA/GA balance (Figure 2b), upregulated energy metabolism and material transport-related genes, and activated the phenylpropanoid biosynthesis pathway (Figure 2a). These gene expression changes are consistent with the physiological process of seed germination [19], and supplementally explain the internal expression characteristics of the reagent combination promoting immature grain germination. However, the above results are only preliminary transcriptional level analysis, and the specific physiological and biochemical changes in immature grains under the reagent combination need to be further verified in subsequent studies.

### 3.2. Immature Grain Germination Technology Has Important Practical Value for Wheat Speed Breeding

The core of wheat speed breeding is to shorten the breeding cycle; advancing the harvest time of grains and accelerating the germination of immature grains are the key technical links. This study selected 18 dpa as the harvest time of immature winter wheat grains, which not only ensures the structural integrity of the embryo and the basic germination potential of grains, but also advances the harvest time by about 7 days compared with the traditional 25 dpa in speed breeding. Combined with the technical characteristic that the germination rate of WGH combination-treated grains peaks at 4 days after treatment, sowing can be carried out 3–4 days after grain harvest and drying, which further compresses the “harvest–germination–planting” cycle. This technical protocol is simple and operable, suitable for greenhouse recurrent selection and generation advancement breeding, and provides a reliable technical operation plan for realizing the “five generations per year” speed breeding of winter wheat.

Different wheat ecotypes have significant differences in embryo development and physiological maturity, and the germination rate of 15 dpa immature grains of winter wheat KN199 in this study was extremely low (<10%), which is very different from spring wheat that can achieve 80% germination rate at 2 weeks after anthesis [20]. This study targeted the physiological characteristics of winter (facultative winter) wheat, selected 18 dpa as the optimal harvest time, and screened out the matching reagent combination, which makes the germination technology highly targeted and applicable to winter wheat. This technology is an important supplement and optimization of the existing wheat speed breeding technology system, which solves the technical problem of low germination rate of immature winter wheat grains and expands the application scope of speed breeding technology in winter wheat genetic improvement.

## 4. Materials and Methods

### 4.1. Grain Drying

The winter wheat cultivar KN199 was grown in the greenhouse of the Institute of Crop Sciences, Chinese Academy of Agricultural Sciences, with a 18 h photoperiod. The flowering date of each spike was recorded when one-third of its spikelets reached anthesis. At 18 dpa, the spikes were harvested and dried at 37 °C for 24 h, manually threshed, and the grains were further dried for another 12 h for subsequent germination experiments.

### 4.2. Grain Germination and Reagent Screening

Dried grains were surface-sterilized with 5% sodium hypochlorite for 15 min and rinsed three times with ultrapure water. Twenty sterilized grains were placed in a 6 cm × 6 cm Petri dish lined with filter paper, and 10 mL of a test solution was added (ultrapure water as control). The test reagents included 1% H_2_O_2_ (CAS No. 7722–84–1, Macklin, China), 20 μM GA_3_ (CAS No. 77–06–5, Macklin, Shanghai, China), and 0.2 mM, 0.5 mM, 1 mM sodium tungstate (Na_2_WO_4_, CAS No. 13472–45–2, Macklin, China), with single, two and triple combinations set as treatments (Table S1). Following the method of [15], each treatment had three independent biological replicates (20 grains per Petri dish).

The Petri dishes were placed in a 30 °C growth chamber for 24 h imbibition, then rinsed three times with ultrapure water and transferred to a 25 °C chamber with a 16 h light/8 h dark cycle. Germination was defined as the visible protrusion of the radicle through the seed coat, and germinated grains were counted daily for 7 consecutive days (the 24th hour after imbibition was designated as Day 1). The germination rate was calculated as: (cumulative number of germinated grains/total number of grains) × 100%. Three independent biological replicates were set for each treatment, and one-way ANOVA was used to test the significant differences in germination rates among treatments by SPSS v27 software.

### 4.3. Transcriptome Sequencing and Analysis

Total RNA was extracted from the germinated grains of control, GH and WGH groups using the Vazyme RC411–01 kit (Vazyme Biotech Co., Ltd., Nanjing, China), and transcriptome libraries were constructed with the Vazyme NR605 kit (Vazyme Biotech Co., Ltd., Nanjing, China) and sequenced on the Illumina NovaSeq 6000 platform (Illumina, Inc., San Diego, CA, USA). The clean data volume of each sample was not less than 95% of the target 10 Gb). Germinated seeds were collected 24 h after the start of imbibition when the radicle protruded throughe seed coat and immediately stored in liquid nitrogen for subsequent RNA extraction. Three independent biological replicates were set for each treatment.

Clean reads were aligned to the Chinese Spring reference genome (IWGSC RefSeq v1.1), and gene expression levels were quantified by FPKM method. DEGs were identified with the thresholds of FDR < 0.05 and |log2(fold change)| ≥ 1. Functional annotation and enrichment analysis of DEGs were conducted via the TGT online platform (https://www.bioinformatics.com.cn/) for gene ontology (GO) terms and the Kyoto Encyclopedia of Genes and Genomes (KEGG) pathways.

## 5. Conclusions

This study identified the optimal reagent combination (1% H_2_O_2_ + 20 μM GA_3_ + 0.5 mM Na_2_WO_4_) for promoting the germination of immature winter wheat grains, achieving a germination rate of 80%. Transcriptome analysis indicated that this combination works by regulating the expression of key genes involved in ABA/GA balance, enhancing secondary and energy metabolism, thereby synergistically driving seed germination. This established technique advances the grain harvest time to 18 dpa, and sowing can be conducted after just 3–4 days of treatment, significantly shortening the breeding cycle. It is well-adapted to winter (facultative winter) wheat, thereby supplementing and optimizing the existing speed breeding system and providing practical technical support for wheat genetic improvement.

## Data Availability

The raw RNA-seq data generated in this study have been deposited in ScienceDB (Agriculture Branch), available for download at https://doi.org/10.57760/sciencedb.agriculture.00280.

## Supplementary Materials

The following supporting information can be downloaded at: https://www.mdpi.com/article/10.3390/plants15091313/s1. Figure S1: Principal Component Analysis (PCA) plot of transcriptomic sequencing data; Figure S2: Volcano plot of differentially expressed genes (DEGs) under GH and WGH treatments; Figure S3: Significantly enriched Gene Ontology (GO) terms of DEGs under GH and WGH treatments; Figure S4: LEA genes down-regulated under both GH and WGH treatments; Figure S5: Significantly enriched GO terms and KEGG pathways of DEGs specifically regulated under WGH treatment; Table S1: Germination treatments for immature wheat grains collected 18 days post anthesis; Table S2: Summary of RNA-sequencing data from germinated wheat seeds; Table S3: Alpha-amylase genes up-regulated under both GH and WGH treatment. Reference [21] is cited in the Supplementary Materials.
