# Supplementary material for: H2O2-GA3-Na2WO4 Synergistically Promotes Germination of Immature Winter Wheat Grains for Speed Breeding
Source: Plants (Basel). 2026 Apr 24;15(9):1313. doi: 10.3390/plants15091313 (PMC13165271; doi:10.3390/plants15091313)
Supplement: Supplementary file 1 [file plants-15-01313-s001.zip › plants-4201431-supplementary.pdf]

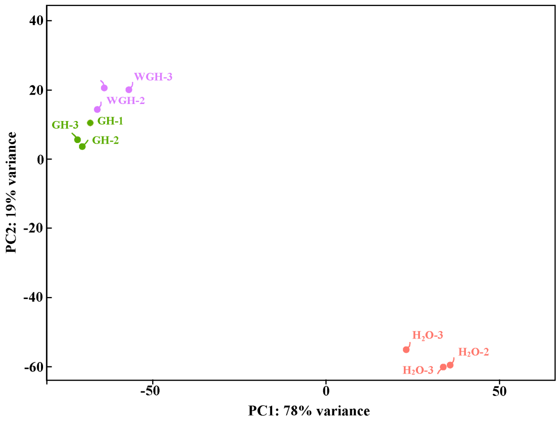

Figure S1. Principal Component Analysis (PCA) plot of transcriptomic sequencing data. Samples were grouped by treatment: WGH (Na_2WO_4 + GA_3 + H_2O_2, purple), GH (GA_3+ H_2O_2, green), and H_2O (water control, red), with three biological replicates for each treatment. The first principal component (PC1) and second principal component (PC2) account for 78% and 19% of the total variance, respectively.

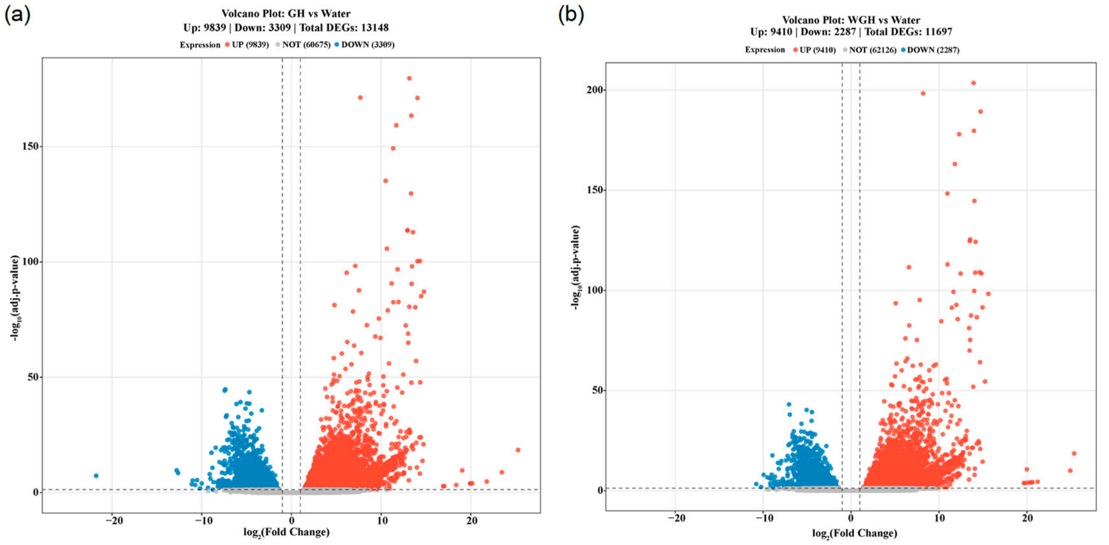

Figure S2. Volcano plot of differentially expressed genes (DEGs) under GH and WGH treatments.

(a) DEGs in the GH vs H_2O comparison; (b) DEGs in the WGH vs H_2O comparison. Red, blue and gray dots represent upregulated, downregulated and non-significant genes, respectively. The x-axis represents log_2(fold change), and the y-axis represents $-log_{10}$(adjusted *p*-value). Dashed lines indicate significance thresholds ($|log_2FC| \geq 1$, adjusted p-value < 0.05).

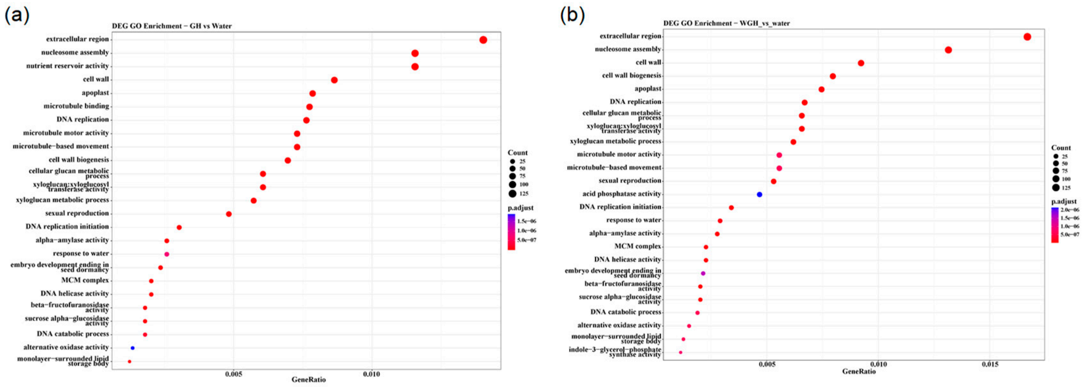

Figure S3. Significantly enriched Gene Ontology (GO) terms of DEGs under GH and WGH treatments.

(a) Top 25 enriched GO terms for DEGs in the GH vs H_2O comparison; (b) Top 25 enriched GO terms for DEGs in the WGH vs H_2O comparison. Dot size indicates the number of genes enriched in the each GO term, and the color gradient indicates the enrichment significance (adjusted *p* value).

(a)

Gene ID	GH	WGH	*LEA* classification[1]
TraesCS2A02G449700	-5.26	-4.66	*TaLEA1-1*
TraesCS2A02G449800	-2.43	-2.15	*TaLEA1-2*
TraesCS2B02G471500	-7.17	-5.91	*TaLEA1-17*
TraesCS2B02G471600	-4.10	-3.89	*TaLEA1-18*
TraesCS2D02G449200	-4.04	-3.19	*TaLEA1-23*
TraesCS2D02G449300	-3.58	-3.32	*TaLEA1-24*
TraesCS4A02G445800	-4.23	-2.51	*TaLEA1-4*
TraesCS5A02G172800	-4.59	-4.34	*TaLEA1-6*
TraesCS5B02G170200	-4.83	-4.96	*TaLEA1-19*
TraesCS5D02G177300	-5.20	-5.45	*TaLEA1-25*
TraesCS6A02G215300	-4.12	-3.99	*TaLEA1-7*
TraesCS6B02G244900	-3.04	-3.12	*TaLEA1-20*
TraesCS6D02G197600	-3.04	-3.20	*TaLEA1-26*
TraesCS7A02G042500	-2.97	-1.71	*TaLEA1-10*
TraesCS7A02G226900	-5.90	-5.75	*TaLEA1-15*
TraesCS7A02G439200	-2.94	-2.75	*TaLEA1-16*
TraesCS7B02G192700	-5.65	-5.89	*TaLEA1-21*
TraesCS7B02G337800	-4.63	-4.64	*TaLEA1-22*
TraesCS7D02G059600	-2.75	-2.09	*TaLEA1-32*
TraesCS7D02G227500	-4.68	-4.17	*TaLEA1-34*
TraesCS7D02G428800	-3.67	-3.33	*TaLEA1-35*

(b)

Gene ID	GH	WGH	*DHN* classification[2]
TraesCS3A02G254600	-4.75	-4.85	*dehydrin Rab25*
TraesCS3B02G286600	-4.02	-3.87	*dehydrin Rab25*
TraesCS3D02G255500	-6.15	-5.98	*dehydrin Rab25*
TraesCS4A02G250900	-3.32	-3.26	*dehydrin DHN4*
TraesCS4B02G064200	-3.51	-2.99	*dehydrin DHN4*
TraesCS4D02G063100	-3.79	-3.73	*dehydrin DHN4*
TraesCS5A02G369900	-4.45	-4.29	*dehydrin DHN1*
TraesCS5B02G372200	-3.50	-3.63	*dehydrin DHN1*
TraesCS5D02G379300	-3.64	-3.33	*dehydrin DHN1*
TraesCS5A02G424700	-3.28	-3.25	*dehydrin Rab15*
TraesCS5A02G424800	-3.19	-3.28	*dehydrin Rab15*
TraesCS5B02G426700	-4.69	-5.56	*dehydrin Rab15*
TraesCS5B02G426800	-3.26	-3.06	*dehydrin Rab15*
TraesCS6A02G059800	-4.91	-4.92	*dehydrin Rab15*
TraesCS6A02G350500	-3.62	-3.57	*dehydrin DHN4*
TraesCS6B02G383500	-3.08	-2.81	*dehydrin DHN4*
TraesCS6D02G332900	-3.49	-3.16	*dehydrin DHN4*

Figure S4. LEA genes down-regulated under both GH and WGH treatments.

(a) Genes belonging to the LEA1 family; (b) Dehydrin-annotated genes (LEA2 family).

Note: [1] LEA gene classification according to [21]. [2] Gene annotation was performed via blastx search against the NCBI database. Values represent $\log_2$(fold change).

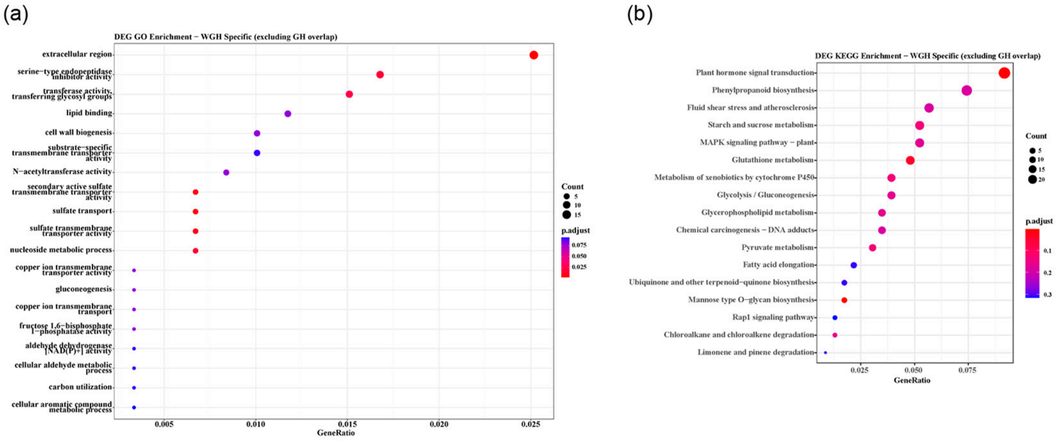

Figure S5. Significantly enriched GO terms and KEGG pathways of DEGs specifically regulated under WGH treatment.

(a) Top significantly enriched GO terms for WGH-specific DEGs (DEGs in WGH vs H_2O after excluding overlapping DEGs in GH vs H_2O); (b) Top significantly enriched KEGG pathways for WGH-specific. Dot size represents the number of genes enriched in each term/pathway, and color indicates the adjusted p-value.

Table S1. Germination treatments for immature wheat grains collected 18 days post anthesis

Test 1	Test 2	Test 3	Control
A	A + B	A + B + C	
B	A + C	A + B + D	
C	A + D	A + B + E	
D	A + E		H_2O
E	B + C		
	B + D		
	B + E		

Treatment codes: A, 1% H_2O_2; B, GA_3 (20 µM); C, Na_2WO_4 (0.2 mM); D, Na_2WO_4 (0.5 mM); E, Na_2WO_4 (1 mM); H_2O_2 (CAS No. 7722-84-1), GA_3 (CAS No. 77-06-5), sodium tungstate (Na_2WO_4, CAS No. 13472-45-2)

Table S2. Summary of RNA-sequencing data from germinated wheat seeds

Reagent (s)	Clean reads	Clean bases	GC Content	%≥Q30	Mapped Reads[a]
H_2O-1	88,971,656	13,307,949,432	57.53%	94.49%	84,398,512 (94.86%)
H_2O-2	94,800,874	14,121,527,908	58.09%	95.41%	89,994,469 (94.93%)
H_2O-3	88,768,324	13,234,491,220	57.42%	95.41%	84,090,233 (94.73%)
GH-1	82,770,032	12,340,323,366	58.00%	95.35%	80,402,809 (97.14%)
GH-2	100,191,100	14,847,123,644	57.36%	95.95%	97,395,768 (97.21%)
GH-3	82,017,410	12,084,810,278	57.62%	95.93%	79,622,501 (97.08%)
WGH-1	75,708,408	11,190,169,314	59.58%	96.39%	73,104,038 (96.56%)
WGH-2	109,789,380	16,358,903,218	57.96%	95.37%	106,254,161 (96.78%)
WGH-3	104,337,756	15,395,861,838	59.18%	96.73%	100,341,619 (96.17%)

[a], sequenced reads are mapped to Chinese Spring reference genome V1.1

Table S3. Alpha-amylase genes up-regulated under both GH and WGH treatment

Gene ID	GH	WGH
TraesCS6A02G319300	13.0	13.5
TraesCS6A02G334100	13.1	13.6
TraesCS6A02G334200	13.5	14.1
TraesCS6B02G349500	14.1	14.6
TraesCS6B02G349700	14.3	14.8
TraesCS6B02G349800	13.0	13.5
TraesCS6B02G364800	13.4	14.0
TraesCS6B02G364900	13.8	14.3
TraesCS6D02G298500	13.3	14.0
TraesCS6D02G313300	13.4	13.9
TraesCS6D02G313500	13.0	13.5
TraesCS7A02G383200	13.4	14.0
TraesCS7A02G383900	14.1	14.7
TraesCS7A02G384000	5.3	5.6
TraesCS7B02G286000	12.9	13.5
TraesCS7B02G286100	14.5	14.9
TraesCS7B02G286700	13.2	13.9
TraesCS7D02G379700	11.8	12.5
TraesCS7D02G380400	11.8	12.5
TraesCS7D02G380500	11.9	12.1

Values are log_2(FoldChange). Red indicates upregulation compared with the water control. WGH treatment consists of 0.5 mM Na_2WO_4, 20 μM GA_3 and 1% H_2O_2, while GH consists of 20 μM GA_3 and 1% H_2O_2.
